# Detection of alveolar bone defects with three different voxel sizes of cone-beam computed tomography: an *in vitro* study

**DOI:** 10.1038/s41598-019-44675-5

**Published:** 2019-05-31

**Authors:** Ting Dong, Lingjun Yuan, Lu Liu, Yifeng Qian, Lunguo Xia, Niansong Ye, Bing Fang

**Affiliations:** 1 Department of Orthodontics, Ninth People’s Hospital Affiliated to Shanghai Jiao Tong University, School of Medicine, Shanghai, China; 2 Department of Oral and Cranio-maxillofacial Science, Ninth People’s Hospital Affiliated to Shanghai Jiao Tong University, School of Medicine, Shanghai, China

**Keywords:** Oral diseases, Preclinical research

## Abstract

This study was conducted to assess the accuracy of cone-beam computed tomography (CBCT) of different voxel sizes in the detection of alveolar bone defects, and to select the optimal voxel size for clinical use. 46 *in-vitro* teeth were placed in bovine ribs in which alveolar bone defects were randomly simulated. In total, 32 alveolar bone defects and 14 teeth without periodontal defects were used. CBCT images were acquired with the use of three different voxel sizes: 0.125-mm, 0.2-mm and 0.4-mm. The scan data were 3D-reconstructed in Mimics software and evaluated by two observers with more than 5 years of experience in CBCT. Receiver operating characteristic (ROC) curves and diagnostic values were obtained. Pairwise comparison of ROC curves was made for evaluation of the diagnostic values of different voxel sizes. Kappa statistics assessed the observer reliability. Results were considered significant at *P* < 0.05. It showed no statistically significant difference between 0.125-mm group and 0.2-mm group, but 0.4-mm group had lower Az values that differed significantly from 0.125-mm and 0.2-mm groups (*P* < 0.05). Based on diagnostic value and radiation protection, 0.2-mm voxel size may be a good choice for the detection of bone defects with CBCT.

## Introduction

In the last years, CBCT has achieved wide acceptance in craniomaxillofacial imaging, which enables three-dimensional evaluation of the jaws and teeth, is superior to digital radiography due to its absence of the anatomical limitations or geometric imperfections^1^.

However, CBCT has the drawback of having a higher radiation dose compared to conventional radiography frequently used in clinics^2^. Effective radiation, the sum of the radiation dose received by all irradiated tissues, represents a potential risk to health including the possibility of carcinogenesis and genetic damages^3^. Even if the probability is very low, CBCT does have the risk of causing damage to DNA molecules due to ionization, especially for young children. It is estimated that children may be two to ten times prone to radiation-induced carcinogenesis than adults^4^.

Therefore, CBCT should be indicated with the criteria only after the clinical examination has been performed and only when the benefits for the diagnosis and treatment planning exceed the risks of a greater radiation dose. To achieve this, the field of view (FOV) should be restricted as much as possible and the resolution should be set as low as possible without damaging evaluation of the area of interest^5^.

The quality of images obtained by CBCT depends on many acquisition parameters such as tube voltage, tube current, FOV, and voxel size^6^. Voxel size is of paramount importance in terms of scanning and reconstruction times, and quality of CBCT images^7^. There are many studies in the literature regarding the effects of voxel sizes on CBCT images. Wenzel *et al*.^8^ used the clinical truth as the gold standard and concluded that high-resolution original CBCT images (0.125 mm) had higher sensitivity than lower-resolution images (0.25 mm) and PSP (photostimulable storage phosphor) images. Melo *et al*.^9^ demonstrated that lower-resolution images (0.3-mm) were not a reliable protocol for the investigation of longitudinal root fractures. On the contrary, da Silveira *et al*.^10^ compared the ability of CBCT examinations with different voxel sizes to detect vertical root fractures in extracted teeth with or without root canal treatment and metallic posts and found similar specificity, sensitivity, and accuracy for both 0.2 and 0.3-voxel resolution scans for unfilled teeth. Regarding 3D reconstruction, Ye *et al*.^11^ discovered that the volume measurements of teeth tended to be larger with increasing voxel sizes during scanning (laser scanning as the gold standard). Yan-Hui Sang *et al*.^12^ found that increasing voxel resolution from 0.30 to 0.15 mm does not result in increased accuracy of 3D tooth reconstruction (3Shape optical scanning as the gold standard).

ALARA,^13^ the acronym used in radiation safety (“As Low As Reasonably Achievable”), means making every reasonable effort to maintain exposures to ionizing radiation as far below the dose limits as practical and has been widely accepted in clinical use. Images acquired in smaller voxel sizes will increase the radiation dose to the patient but might provide the same diagnostic outcome as lower-resolution images, so we must choose optimal voxel size based on the reliability and accuracy of the diagnostic outcome and radiation dose. This study aimed to assess the effect of different voxel resolutions on the accuracy of diagnosis with CBCT, to achieve a balance between radiation dose and diagnostic accuracy. Due to the ALARA principle, we adopted an *in vitro* experiment as the preliminary investigation.

## Materials and Methods

### Sample establishment

**Figure 1:**
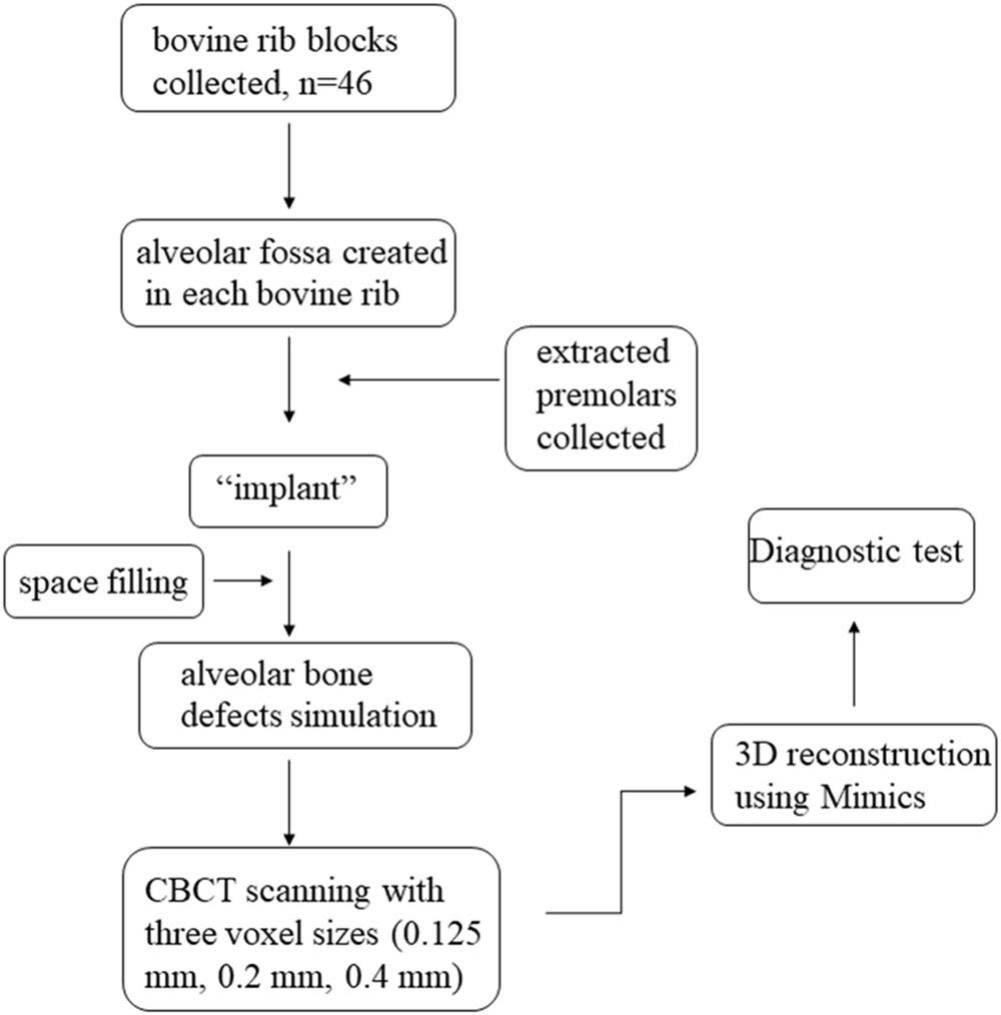
A schematic drawing of the study.

**Figure 2:**
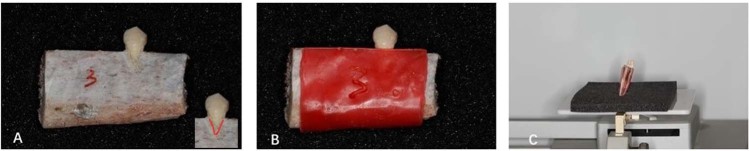
CBCT scan of bovine rib covered by boxing wax. (**A**) Dehiscence created on the bovine rib. The defect was indicated with red marker. (**B**) Bovine rib covered by boxing wax. (**C**) Scan the sample with CBCT.

A schematic drawing was created to show the process of this study clearly (Fig. 1). Forty-six bovine rib blocks were used to simulate the alveolar bone in this study. The rib ridge was flattened, and an alveolar fossa was created in each bovine rib by means of burrs fixed on a high-speed handpiece, after which an extracted premolar was ‘implanted’ into the alveolar fossa. Bovine bone powder from rib ridge trimming was mixed with plaster powered in a ratio of 7:3 and then some water was added to produce a pasty mixture. The narrow space between the tooth and bone was filled with the mixture. The bone defects were artificially created on an edge of the bovine rib with spherical and cylindrical burrs, which correspond to the cervical portion of the implanted tooth. According to Evangelista *et al*.^14^, at least three consecutive sections of CBCT images showing no bone coverage on the root surface can be identified as an alveolar bone defect, and the defect of at least 2 mm below the enamel-dentinal junction can be identified as fenestration. These defects were created 3 mm wide and 4 mm in length, taking the research of Bagis *et al*.^15^ as a reference (Fig. 2). This study was approved by the Institutional Review Board of Shanghai Ninth People’s Hospital affiliated to Shanghai Jiao Tong University, School of Medicine. It is confirmed that all experiments were performed in accordance with relevant guidelines and regulations. The ribs with the teeth were covered by double layers of boxing wax (Dental material factory of Shanghai medical international Co. LTD) for soft tissue simulation.

### CBCT acquisition and 3D reconstruction

CBCT acquisition was performed with a KaVo 3D exam scanner (KaVo Dental GmbH, Biberach, Germany) with a field-of-view size (FOV) of 8.5 × 8.5 cm, 120 kv, and 5 mA. Three voxel sizes were used: 0.125 mm, 0.2 mm, and 0.4 mm. The filler was also scanned by CBCT to confirm that the CT Hounsfield unit (HU) was within the CT value of the cortical bone and reconstruction threshold. The final sample was comprised of 32 blocks for the evaluation of alveolar bone defects, and 14 blocks as controls.

**Figure 3:**
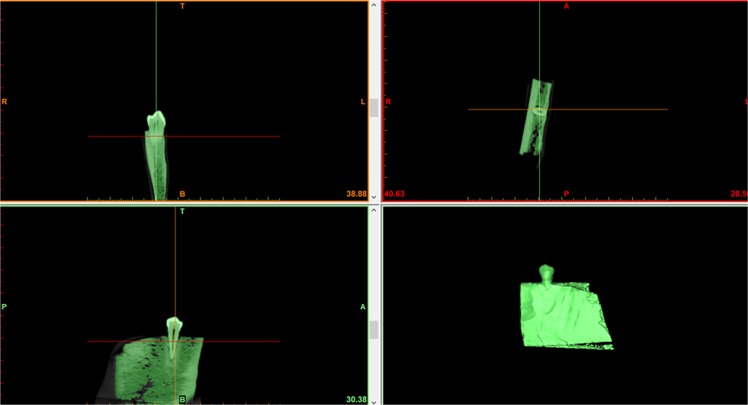
Three-dimensional reconstruction with the software Mimics. The image shown was obtained with 0.125 mm voxel size.

**Figure 4:**
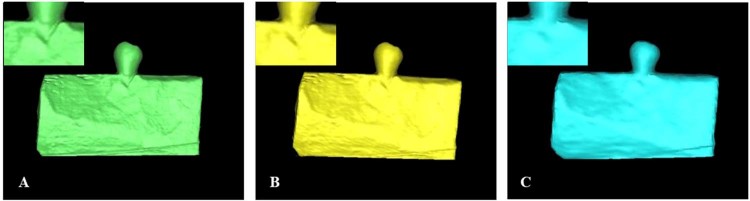
Different 3D reconstruction of the same dehiscence sample with three voxel sizes. The defects were enlarged and highlighted with red marker. (**A**) 0.125 mm; (**B**) 0.2 mm; (**C**) 0.4 mm.

All images were saved in DICOM format and exported to Mimics 10.01 software (Materialise, Leuven, Belgium). The images were three-dimensionally reconstructed with the maximum threshold of 2876 HU and the minimum threshold of 200 HU (Fig. 3)^16^. Then the calculated 3D model was exported in STL file format (Fig. 4).

### Diagnostic evaluation

Two clinicians with at least five years of experience in CBCT blindly evaluated all the images in 3Shape 3D viewer software, version 3.0.34 (3Shape, Copenhagen, Denmark). The time allocated for the observations was not restricted. The evaluation adopted a dichotomous scale for the presence/absence of defects (1, absent; 0, present). The images were re-evaluated after 30 days.

### Statistical analysis

IBM SPSS Statistics 20.0 (IBM, Chicago, IL, USA) and MedCalc 18.2.1 (MedCalc Software, Ostend, Belgium) were used for statistical analysis. Intra- and inter-observer agreements were calculated with the use of the kappa test (poor agreement, <0.40; moderate agreement, 0.40–0.59; good agreement, 0.60–0.79; excellent agreement, 0.80–1.00). Receiver operating characteristic (ROC) curves and diagnostic values were obtained. The Az values were compared by analysis of variance.

## Results

Reliability statistics for intra-observer agreement resulted in excellent kappa values (from 0.893 to 0.942), and values for inter-observer agreement ranged from good to excellent (from 0.677 to 0.836).

**Figure 5:**
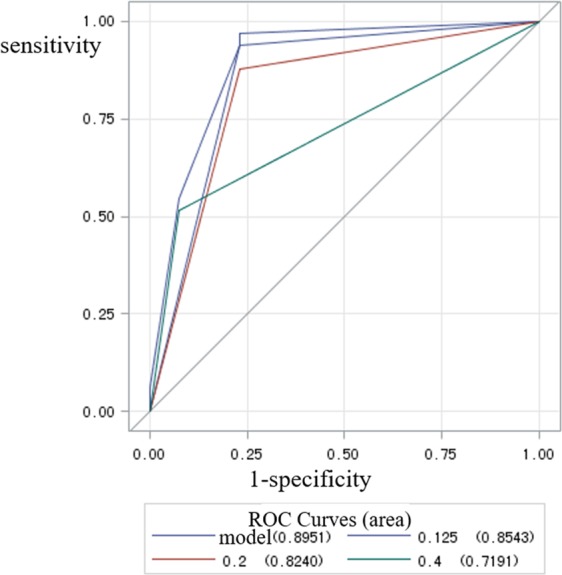
ROC curve of the accuracy of detection of alveolar bone defect at each voxel sizes.

ROC curve was used for data analysis (Fig. 5) and the Az values (Area under the ROC curve) for the observers are summarized in Table 1. There was no statistically significant difference between the 0.125-mm group and the 0.2-mm group. However, the 0.4-mm group had lower Az values than the 0.125-mm and 0.2-mm groups. Table 1 also shows the sensitivity, specificity, positive predictive value (PPV), and negative predictive value (NPV) for the protocols. Sensitivity values and NPV values were highest in the 0.125-mm group and lowest in the 0.4-mm group, while specificity values were highest in the 0.4-mm group and lowest in the 0.125-mm group. Pairwise comparison of Az values for the three protocols is also presented in Table 1. There was no statistically significant difference between the 0.125-mm group and the 0.2-mm group. There was a statistically significant difference between the 0.125-mm group and the 0.4-mm group (*P* < 0.05), and between the 0.2-mm group and the 0.4-mm group (*P* < 0.05).

**Table 1:** Area under the receiver operating characteristic curve (Az values) for the observers in the protocols tested.

Group	Az values	95% CI		Sensitivity (%)	Specificity (%)	PPV (%)	NPV (%)
		lower	upper				
0.125-mm	0.869*	0.727	1.000	96.97	76.92	91.43	90.91
0.2-mm	0.854**	0.709	0.999	93.94	76.92	91.18	83.33
0.4-mm	0.689***	0.531	0.846	45.45	92.31	93.75	40.00

PPV, Positive predictive value; NPV, negative predictive value.

Pairwise comparison of ROC curves:

* and **P = 0.3173.

* and ***P = 0.0209 < 0.05.

** and ***P = 0.0117 < 0.05.

## Discussions

The quality of images obtained by CBCT depends on acquisition parameters such as tube voltage, tube current, the field of view (FOV) and voxel size^7^. Many of these parameters can be varied according to the diagnostic task, but still, no protocols have been established for specific diagnostic tasks in dentistry.

Voxel is the minimum unit of digital data segmentation in three-dimensional space, similar in concept to the pixel in two-dimensional space. There are many studies in the literature regarding the effects of voxel sizes on CBCT images. Digital caliper measurements as the gold standard, Sun and co-authors^17^ showed that 0.4-mm voxel size CBCT images might overestimate alveolar bone-height loss associated with rapid palatal expansion, and these measurement inaccuracies were substantially improved with 0.25-mm voxel size. While Patcas and colleagues^18^ showed that 0.4-mm voxel images provided results not significantly different from those provided by 0.125-mm voxel images. This finding is also in agreement with reports by Torres and co-authors^19^, who found the accuracy of vertical and horizontal measurements was shown to be comparable with the measurements performed on the dry mandible with four voxel sizes. de-Azevedo-Vaz *et al*.^20^ tested the effect of voxel sizes (0.2-mm and 0.12-mm) and scan modes on peri-implant fenestration and dehiscence detection and concluded that the voxel sizes did not affect fenestration and dehiscence detection.

From our research, we found that 0.125-mm voxel images had the highest diagnostic value based on Az values, while 0.2-mm voxel images had nearly the same values as 0.125-mm. Further, the 0.4-mm voxel images had obviously lower diagnostic value than the 0.125-mm and 0.2-mm images, with much lower sensitivity and PPV values and high false-negative readings. There was no statistically significant difference between the 0.125-mm group and the 0.2-mm group. There was a statistically significant difference between the 0.125-mm group and the 0.4-mm group (*P* < 0.05), and between the 0.2-mm group and the 0.4-mm group (*P* < 0.05). It was shown that the 0.2-mm voxel size had nearly the same high diagnostic value as the 0.125-mm voxel size, but that the 0.4-mm voxel size was not clear enough for accurate detection of bone defects.

The partial-volume effect^11,21^, which is a common artifact in computed tomography, may explain the difference in diagnostic accuracy of different voxels. According to the theory of the partial-volume effect, if a voxel lies completely within an object, it would reflect that object’s density. However, when a voxel lies on the borders of two objects of different densities, this voxel will then reflect the average density of both objects rather than the true value of either object. Therefore, when we use a larger voxel size (e.g., 0.4 mm), the tooth volume will be somewhat larger, and the margin of reconstructed bone will be comparatively vague because of defects somewhat covered by artifacts, possibly explaining why larger voxel sizes have lower diagnostic value.

The use of CBCT remains controversial due to its higher radiation dose compared with two-dimensional images. The radiation dose of CBCT is quite different among different brands and varies due to the different settings of scanning parameters such as tube current, FOV and resolution. Voxel size is a key factor determining the spatial resolution of image. Choosing a smaller voxel size will increase the spatial resolution of image, but it also means more x-rays for patients. The radiation dose of CBCT is positively correlated with exposure time. A smaller voxel size (e.g., 0.125 mm) usually means longer exposure time which inevitably increase the radiation dose^4^. The conflict is between the clinicians’ desire to visualize the anatomical structures of the patient at a high resolution and the risk related to the increased radiation dose. The principle of ALARA^13^ requires to maintain ionizing radiation as low as reasonably achievable, which means choosing the minimum resolution that does not affect diagnostic accuracy. Images acquired in smaller voxel sizes will increase the radiation dose to the patient but might provide the same diagnostic outcome as lower-resolution images, so we must choose optimal voxel size based on the reliability and accuracy of the diagnostic outcome and radiation dose. Also, clinicians need a relevant large field of view in most of cases which excess the small field of view provided by o.125 mm voxel size. Taking both accuracy and radiation dose into account, 0.2-mm voxel size may be good choice for the detection of bone defects with CBCT.

## Limits

The periodontal defect simulations were made with the use of burrs, which can be a limitation of the study since such tools can only simulate relatively regular defects and may not represent the natural structures of the periodontal composition. Also, the size of the defect was set as 3 mm in diameter and 4 mm of length, which may be large compared to the voxel sizes used and may have some effect on the results. Future studies should be conducted with better simulating methods which could be more accordant with the natural creation of the defects. Also, there are inescapable differences between the control group and the naturally thin alveolar bone wall. In reality, it may be even 0.1 mm, but it is nearly impossible to create such a thin bone wall without creating alveolar defects, which may cause some differences from clinical diagnosis and decrease false-positive readings. Further research is needed with better *in vitro* models or clinical studies. Another limitation of the study can be the use of wax for soft-tissue simulation. Various materials can be used for the simulation of soft tissues, such as: water, wax, self-polymerizing resin, acryl, paraffin polyethylene, and plexiglas^22–24^. Wax is the most widely used in the soft-tissue simulation, not there is insufficient evidence concerning its use in CBCT. Thus, further research should be conducted for the evaluation of the methods of soft-tissue simulation in CBCT. Meanwhile, more studies are required for prostheses and filled teeth, which are common in the clinic.

## Conclusions

Based on diagnostic value and radiation protection, a 0.2-mm voxel may be the good choice for the detection of alveolar bone defects with CBCT.
